# Giraffes and hominins: reductionist model predictions of compressive loads at the spine base for erect exponents of the animal kingdom

**DOI:** 10.1242/bio.057224

**Published:** 2021-01-22

**Authors:** Michael Günther, Falk Mörl

**Affiliations:** 1 Institut für Modellierung und Simulation Biomechanischer Systeme, Computational Biophysics and Biorobotics, Universität Stuttgart, Nobelstraße 15, 70569 Stuttgart, Germany; 2 Forschungsgesellschaft für Angewandte Systemsicherheit und Arbeitsmedizin mbH, Biomechanik & Ergonomie, Lucas-Cranach Platz 2, 99097 Erfurt, Germany

**Keywords:** Biomechanics, Intradiscal pressure, Frustum, Morphometry, Homo sapiens, Australopith

## Abstract

In humans, compressive stress on intervertebral discs is commonly deployed as a measurand for assessing the loads that act within the spine. Examining this physical quantity is crucially beneficial: the intradiscal pressure can be directly measured *in vivo* in humans, and is immediately related to compressive stress. Hence, measured intradiscal pressure data are very useful for validating such biomechanical animal models that have the spine incorporated, and can, thus, compute compressive stress values. Here, we use human intradiscal pressure data to verify the predictions of a reductionist spine model, which has in fact only one joint degree of freedom. We calculate the pulling force of one lumped anatomical structure that acts past this (intervertebral) joint at the base of the spine, lumbar in hominins, cervical in giraffes, to compensate the torque that is induced by the weight of all masses located cranially to the base. Given morphometric estimates of the human and australopith trunks, respectively, and the giraffe's neck, as well as the respective structures’ lever arms and disc areas, we predict, for all three species, the compressive stress on the intervertebral disc at the spine base, while systematically varying the angular orientation of the species’ spinal columns with respect to gravity. The comparison between these species demonstrates that hominin everyday compressive disc stresses are lower than those in big quadrupedal animals. Within each species, erecting the spine from being bent forward by, for example, thirty degrees to fully upright posture reduces the compressive disc stress roughly to a third. We conclude that erecting the spine immediately allows the carrying of extra loads of the order of body weight, and yet the compressive disc stress is lower than in a moderately forward-bent posture with no extra load.

## Abstract (precis)

**Summary:** Using a simple biomechanical model, we predict the compressive stress on vertebrates’ intervertebral discs loaded by all cranial masses being held anywhere between fully upright and horizontal bow.

## INTRODUCTION

In the extant animal kingdom, a permanently upright (fully erect) posture of the whole spinal column is a rare exception, namely, performed solely by *Homo sapiens*. By ‘upright’ or ‘fully erect’, we mean the alignment of the spine with gravity in a terrestrial way of life. Immediately connected to the notion of upright posture is that the weight of all cranially located body parts may be the crucial factor that determines the mechanical loads on the spine. Accordingly, the focus of this paper is on examining the loads at the base of the spine, where the weights accumulate. In humans, this is the intervertebral disc (IVD) at lumbar level L4/L5.

A measure of mechanical loads on the spine is the compressive stress on an IVD, exerted through the endplates of the adjacent vertebrae. This measure has at least two advantages: first, in humans, measured data of intradiscal pressure are available (Nachemson, 1960, 1966; Brinckmann and Grootenboer, 1991; Sato et al., 1999; Wilke et al., 1999; Takahashi et al., 2006). These data strongly correlate to compressive stress (external pressure) on IVDs (Nachemson, 1960; Brinckmann and Grootenboer, 1991). Second, stress and pressure allow comparisons across body dimensions and species. In this paper, we put the available human data of everyday values of compressive stress the whole IVD is exposed to into context of the animal kingdom. This is as internal loads definitely guide the mechanical design of animals, for example, the base angle setting of leg joints (Biewener, 1989), which entails functional implications to be reflected in terms of (muscle-) mechanics (Seyfarth et al., 2001; Günther and Blickhan, 2002; Günther et al., 2004) and consequently metabolism (Biewener, 1990).

For comparison, one animal species stands out from the extant terrestrial animal kingdom: giraffes do, likewise, not have leg support for a cranially located part of the spine that contains a significant portion of the body mass. The vertebrae in giraffes, like in all mammals’ spines, are contacting via true IVDs, which consist of an annulus fibrosus and a nucleus pulposus. Thus, a giraffe's neck seems the most adequate counterpart for assessing the loads on the human lumbar spine in a comparative approach, with the base of the giraffe's neck at the C7/T1 level being the analogue to the lumbar L4/L5 region in humans.

As a start, we have implied that the direct effect of body weight is a mechanically plausible key factor that determines spinal loads. However, this only holds in cases where the spine approximately aligns with the direction of gravity. If spinal parts that are only supported at their base are deflected away from upright positioning, then, moreover, a weight-compensating torque is required from structures forming the joint at which the cranial spine parts are suspended at the base: in humans, at the vertebra L5, which is itself attached to S1 and, thus, the pelvis, or, in giraffes, at T1 constituting the terminal of the thoracal spine. Generally, these compensatory torque-generating structures are predominantly ligaments and muscles, which pull past the IVD with their respective lever arms, as well as the IVD itself, and the facet joints. Any force exerted by a structure that pulls past an IVD induces in reaction an additional compressive stress to the IVD, with the added stress crucially depending on the structure's anatomical arrangement (lever arm). Helmuth (1985) had demonstrated in principle, while focusing on australopiths, that these pulling-force-induced contributions have a pronounced impact on compressive lumbar IVD stress at 30 degree forward flexion of australopiths’ spines. Unfortunately, the description of his biomechanical model and his calculations were unintelligible, and the predicted compressive stress values both partly irreproducible and, what is more, about ten times higher in australopiths than in humans, partly due to the then-known values of australopith IVD areas being too small. Also, stress values in forward flexion scenarios were not quantitatively compared with such in fully erect posture. Methodically in line with the mechanical analyses by Helmuth (1985); Alexander (1985); Christian and Dzemski (2007), here, we predict, by a reductionist biomechanical model, the compressive stresses on the base IVDs of the human lumbar spine and the giraffe's neck, respectively, while systematically varying the angular orientation of the spinal columns with respect to gravity. Since the relation between intradiscal pressure and compressive stress is empirically known in humans, we then verify the model predictions of compressive stress, and can prove the model valid. Moreover, we compare values of compressive lumbar stress in humans with those likewise predictable by our model for australopiths. The latter calculations are now based on a recent literature source of an australopith's lower lumbar IVD endplate area, which differs significantly from data available almost four decades ago (Helmuth, 1985; Johansson et al., 1982).

Determining mechanical measurands is a fundamental prerequisite for investigating biological tissue build-up, wear and tear, fatigue, damage, and recovery, that is, the responses of living tissue to mechanical loads, as well as the laws and principles of structural organisation of matter in general. It is also generally a means of searching for evolutionary boundary conditions, rules, and design criteria, most notably when putting these data in context across size scales, i.e. examining allometric relations. In this study, we carve out the (few as we think) parameters, and distinguish and weigh two mechanisms, that essentially determine the compressive loads on base structures of erect body parts, in particular, when the degree of erection (spine or neck inclination) is varied.

### Model Formulation

To calculate the compressive loads on the base IVD of the human lumbar spine and the giraffe's neck, we first computed the masses and centre of mass (CoM) positions of the human head–arms–trunk (HAT) and the giraffe's neck–head (HENE) segment assemblies, respectively, the latter by geometrical modelling. In a second step, these and additional data of the IVDs’ geometrical dimensions are then used in a mechanical model for a load analysis.

### Anthropometry

We estimated the human HAT mass and the position of the HAT's CoM for a male human of 1.75 m height and 75 kg weight, using in-house software *calcman2d* (Hahn, 1993). The distance of the HAT CoM position from the centre of the L4/L5 IVD joint, which approximately coincides with the line connecting the hip joint centres, is *L*_*CoM*,*HAT*_=0.265 m, the HAT mass was estimated as *M*_*HAT*_=51 kg. Dimensions of human vertebrae and IVDs are well documented in the literature. Sagittal depth and frontal width values of lumbar endplates have been determined by Nachemson (1960); Brinckmann and Grootenboer (1991); Gilad and Nissan (1985); Zhou et al. (2000). From these data (Table 1), we calculated the compressed area of the lumbar IVD at level L4/5 by again assuming an elliptic form: *A*_*L*4/*L*5_=15 cm^2^. The (mean) lever arm for pulling structures (muscles and ligaments) past the L4/L5 IVD was extracted from Gilad and Nissan (1985): *R*_*pull*_=0.049 m. This set of human model parameters is used to predict, at any trunk (HAT) angle, the compressive force *F*_*L*4/*L*5,||_ on the human L4/L5 IVD according to Eqn 5, and the corresponding compressive stress *P*_*L*4/*L*5_ (with *A*_*L*4/*L*5_) according to Eqn 6. That is, all these model calculations are in full analogy to predicting the compressive force and stress on the giraffe's neck base, with just the human parameter values replacing the corresponding ones (see Table 1) of the giraffe's neck, which are gathered in the next section.

**Table 1.**
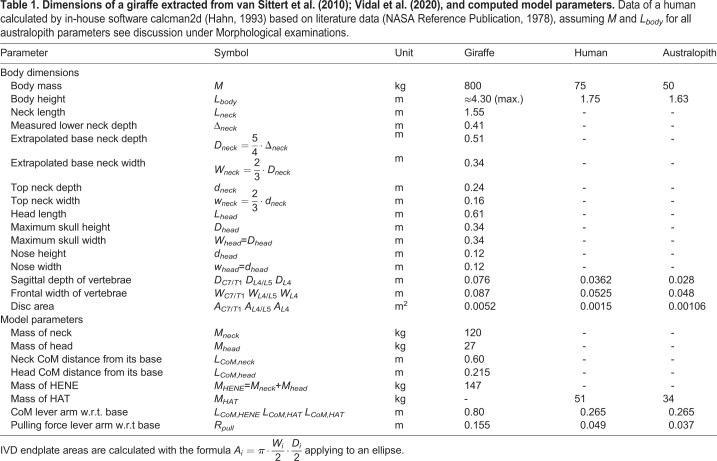
**Dimensions of a giraffe extracted from van Sittert et al. (2010); Vidal et al. (2020), and computed model parameters.** Data of a human calculated by in-house software calcman2d (Hahn, 1993) based on literature data (NASA Reference Publication, 1978), assuming *M* and *L*_*body*_ for all australopith parameters see discussion under Morphological examinations.

### The survey of a giraffe

**Fig. 1.**
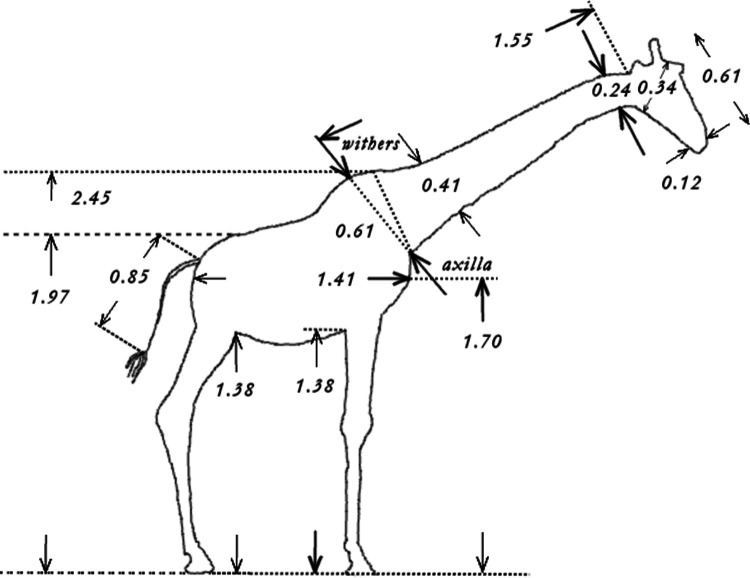
**Geometrical dimensions of an adult (female) giraffe with an overall body length (‘total length’, Mitchell et al., 2009: sum of tail, neck and head lengths, plus distance from tail base to withers) of about 4.30 m and weight of 800 kg, respectively, with the dimensions given in metres.** The neck length is 1.55 m, and the ‘total height’ (Mitchell et al., 2009) is the withers height (2.45 m) plus neck length (1.55 m) plus half of the head height (0.34 m), i.e. 4.17 m. These numbers have been measured in the original drawing of Vidal et al. (2020, Fig. 1). Our sketch here is a freehand drawing, so the numbers may slightly deviate from the distances within our sketch.

We computed the masses and CoM positions of a giraffe's (*Giraffa camelopardalis*) neck and head by modelling them each as an elliptic and circular frustum, respectively. By combining two different sources in the literature, we related the dimensions of neck, head, and other body parts in giraffe's sagittal plane to body mass. Mass and overall body length from the nose to the tip of the tail (without the tassels) have been documented (van Sittert et al., 2010) for thirty-nine young and adult individuals. As a basis for our computations, we measured off the dimensions depicted in Fig. 1 from the contour drawing of a giraffe in figure 1 of Vidal et al. (2020), including lengths and depths of the neck and the head at different body locations. These dimensional data were calibrated by the body height of a true-to-scale contour of a human depicted in the same figure in Vidal et al. (2020) given to be 1.70 m. With all this, we estimated that the giraffe contour in figure 1 of Vidal et al. (2020) represents a female specimen with an overall body length (‘total length’, Mitchell et al., 2009) of about 4.30 m and, thus (van Sittert et al., 2010, Table 1), weight of about 800 kg. For a 800 kg female, a cross-check using the ‘total height’ (Mitchell et al., 2009) measure reveals that our number (Fig. 1: 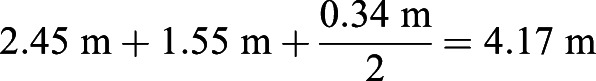) is close to what can be read from Mitchell et al. (2009, Fig. 1A) (4.30 m).

Dimensions in the frontal plane of a giraffe are not documented in the literature. As an alternative, photos from the internet provided us with a rough guess of neck and head widths: we estimated the ratio of frontal width to sagittal depth values of the neck being 2–3, which enabled us to approximate the neck geometry by a frustum with an elliptic base area. For the head, we assumed sagittal depths and frontal widths in a cross-section to be approximately the same, with its geometry hence being roughly representable by a frustum with a circular base area. All parameters are summarised in Table 1.

To predict values of compressive stress (external pressure) on the IVD at the giraffe's cervical spine level C7/T1, depth and width values of the endplates were taken from van Sittert et al. (2010, Fig. 2C,D) (Table 1), and used to calculate the endplate areas assuming that half of the depth and width, respectively, represent the half-axes of an ellipse. They also documented lengths of spinous processes. Based thereon, we set the (mean) lever arm for structures (like the nuchal ligament) that exert pulling forces past the C7/T1 IVD in a giraffe's neck to the endplate depth of T1 plus half of its spinous process length (van Sittert et al., 2010, Fig. 2E), i.e. *R*_*pull*_=0.155 m.

### CoM position of a giraffe's HENE: combining two frustums

**Fig. 2.**
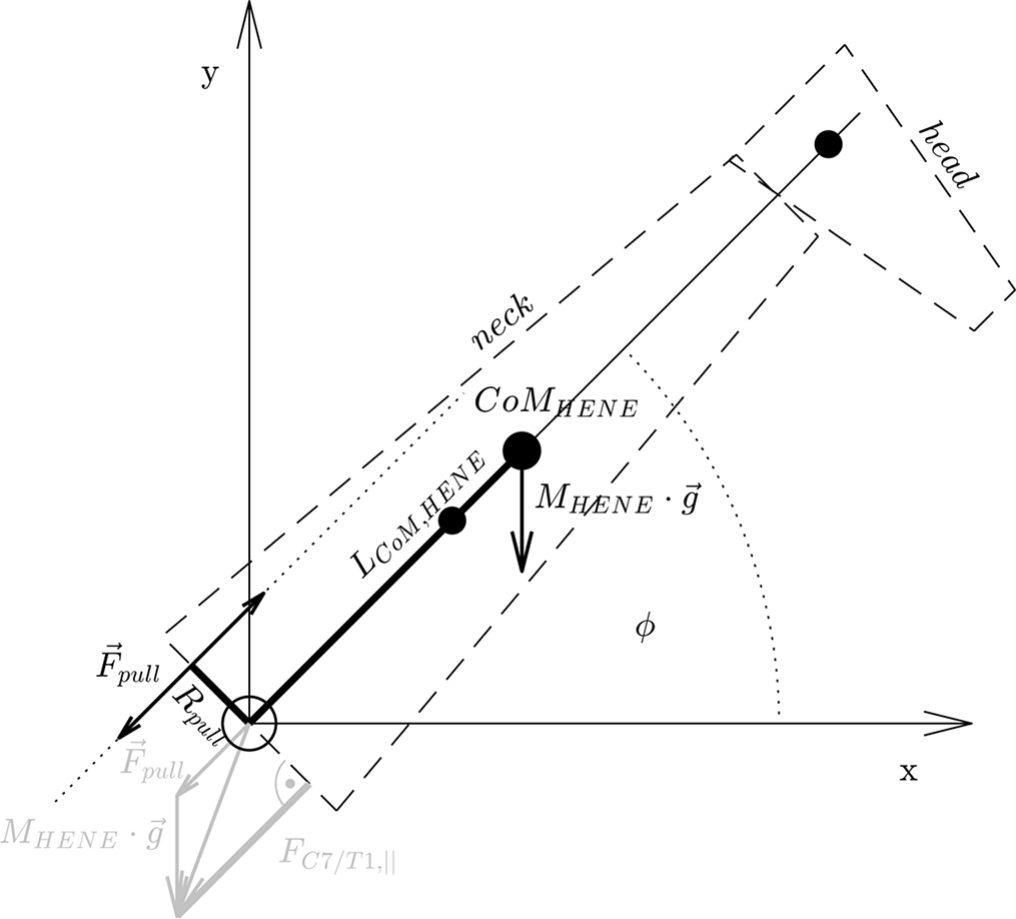
**The geometry and mechanical structure of the model of a giraffe's head-neck (HENE) segment assembly.** True to scale in all dimensions (Table 1), and the modelled forces acting on HENE (condensed in grey at the bottom: only schematically, *F⃗*_*pull*_ is plotted much too short in relation to weight vector). The smaller filled circles are the CoMs of neck and head parts, the thick one is HENE's overall CoM, with the thick black line depicting its distance (Eqn 2) from the base of HENE (at the C7/T1 joint: open circle), and the thin line the head's distance. The thick short line labelled with *R*_*pull*_ and oriented perpendicular to the double arrow that symbolises the pulling force vector *F⃗*_*pull*_ is the lever arm of the *F*_*pull*_-generating structure that spans HENE's base joint and, therewith, compensates the torque (Eqn 4) around the joint generated by HENE's weight *M*_*HENE*_ · *g*. The compressive force *F*_*C*7/*T*1,||_ (Eqn 3 or Eqn 5, respectively) on HENE's base is also depicted: the grey thick line perpendicular to the dashed base line (surface) of HENE, i.e., the projection of the force vector *F⃗*_*pull*_ + *M_HENE_* · *g⃗* on HENE's longitudinal axis. For calculating compressive force *F*_*L*4/*L*5,||_ on a hominin L4/L5 IVD instead of *F*_*C*7/*T*1,||_, HENE parameters are replaced in Eqns 5 and 6, and the sketch here: only the erect body part's mass and CoM position varies among the species, by the corresponding human- or australopith-like ones (see Table 1).

The CoM position of a giraffe's HENE segment was calculated by assembling the positions of a neck segment, modelled as an elliptic frustum, and the head segment, modelled as a circular frustum (the upper, dashed part of Fig. 2 depicts the HENE assembly). In a frustum with an elliptic base (half-axes *a* and *b*), the area 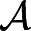 of its base, its volume 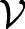, its mass distribution along its longitudinal axis, and, thus, its CoM position on this axis, expressed as distance 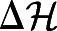 from its base, are the same as in a frustum with a circular base of the equivalent radius 
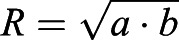
: base area 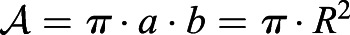 and volume 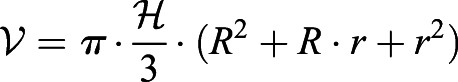. Hereby, 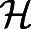 is the height, above the base, of the frustum's smaller, top area of the (equivalent) radius *r*. The CoM position (assuming homogeneous mass density) is (see, e.g. Stöcker, 2008)(1)
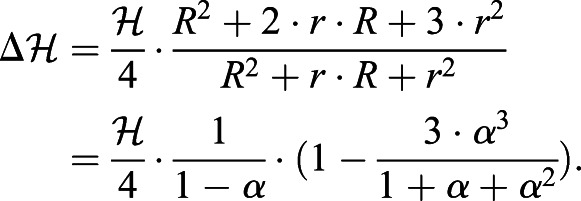
The right equality arises from defining the ratio 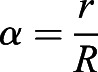, with 0<*α*<1. Given the dimensions in Table 1 and assuming a homogeneous body mass density of approximately water-like *ρ*_*body*_=1000 kg m^−3^, the mass of the giraffe's neck and head segments are calculated as the product of *ρ*_*body*_ and their respective (frustum) volume: approximately *M*_*neck*_=120 kg and *M*_*head*_=27 kg, respectively. The respective CoM distances from their bases are calculated by Eqn 1: *L*_*CoM*,*neck*_=0.60 m and *L*_*CoM*,*head*_=0.215 m, respectively. As a result, the distance of the CoM of the overall HENE segment from the centre of the C7/T1 IVD joint located at its base (in common with the neck segment) is calculated by assembling the neck and head segments:(2)
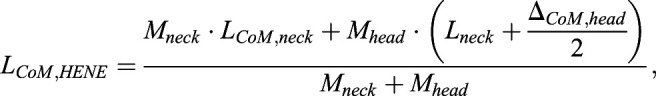
with *M*_*neck*_+*M*_*head*_=*M*_*HENE*_=147 kg being the overall HENE mass, and 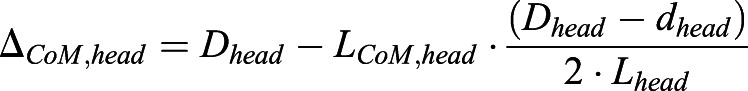 the head depth (height) at the head CoM position: *L*_*CoM*,*HENE*_=0.80 m.

### The compressive load on the base IVD in static equilibrium

We assume that HENE is suspended at a joint located at its base, thinking of a point in the sagittal plane within or at the surface of the loaded C7/T1 IVD (a centre of rotation, force transmission, pressure, or else: the centre of the circle at the base of HENE in Fig. 2). The following calculation of both the bending torque induced by the HENE weight acting around the base joint and its corresponding compensatory, body-internal pulling force (magnitude: *F*_*pull*_; see Fig. 2) exerted by a lumped anatomical structure that passes this IVD joint is analogous to Helmuth (1985), Alexander (1985), and Christian and Dzemski (2007), but we systematically expand this analysis to the whole angular range from fully erect (*φ*=90°) to fully forward bent (flexed: *φ*=0°) HENE postures (*φ*: see Fig. 2). In a giraffe, we primarily think of the lumped anatomical structure to be the nuchal ligament. It is assumed, for simplicity of the model and again in line with Helmuth (1985), Alexander (1985), and Christian and Dzemski (2007), that the structural pulling force vector 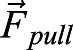 aligns with the longitudinal axis of the HENE segment (Fig. 2), which itself is meant to represent the neck's longitudinal axis. In the *force equilibrium* of the HENE segment, considered in only one dimension, namely, in the direction along the longitudinal axis, the sum of the longitudinal projections of the two forces acting on HENE – the HENE body weight vector 
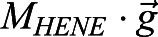
 and the structural force 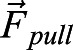 (the magnitude: *F*_*pull*_) pulling HENE to the trunk – are compensated by the corresponding force 
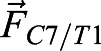
, with *F*_*C*7/*T*1,||_ being the (compressive) projection (see bottom, greyed part of Fig. 2) by which the C7/T1 IVD, placed between HENE and trunk, counters:(3)
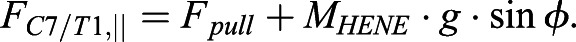
We assume thereby that forces acting in the facet joints are of minor significance in the angle range investigated here (*φ*=0…90°), which seems justified due to the measured range of motion of the C7/T1 joint alone being between 40° (Stevens and Parrish, 2005, Fig. 6.13) and 50° (Dzemski and Christian, 2007, Fig. 9). Note that we do not analyse shear forces in this study. In the most reduced model here, 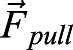 does not induce any shear force, thus, shear force in our model is *M*_*HENE*_ · *g* · cos*φ* : zero, if HENE is fully erect, and HENE weight, if horizontal posture is adopted.

For also fulfilling static *torque equilibrium* of the HENE segment, the torque exerted by 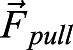 via the lever arm *R*_*pull*_ around the IVD joint compensates the external (bending) torque around this joint exerted by the HENE weight acting on HENE at its CoM:f(4)
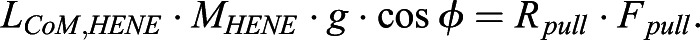
Solving Eqn 4 for *F*_*pull*_ and inserting this into Eqn 3, we find the load the IVD joint has to counter:(5)
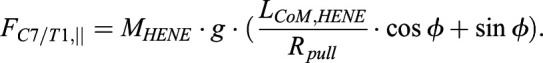
We interpret *F*_*C*7/*T*1,||_ as the compressive load on the C7/T1 IVD, which implies that this load is acting mainly perpendicular (normal) to the compensating structural (IVD) surface. As the anatomy of the cervical joints in giraffes is ball-and-socket-like (van Sittert et al., 2010; Vidal et al., 2020; Danowitz and Solounias, 2015), this implication generally seems well justified in these animals, and referring to a giraffe's C7/T1 joint, ‘shear force’ (see above) is a mathematical rather than a physical term.

For our comparing the giraffes' compressive loads at the base of the neck with those in humans at their lumbar L4/L5 level, we accordingly (i) apply Eqn 5 to humans, with solely exchanging the giraffe's model parameters by correspondingly human-like ones (Table 1). Further, we (ii) imply that the angular orientation of the barely curved human L4/L5 IVD surfaces (sandwiched between the two adjacent vertebrae's endplates) is always approximately perpendicular to the longitudinal spine axis, whatever inclination angle *φ* relative to gravity is analysed. That is, in this study, the orientation of the surface of compressive load analysis is chosen the same, at any angle *φ*, in humans (and australopiths) and giraffes. Only the dashed outline of a giraffe's HENE in Fig. 2 must be replaced in our minds’ eyes by a hominin's HAT outline, with an accordingly changed weight and CoM distance from the IVD joint.

To now predict the compressive stress (external pressure) *P*_*i*_ applied to any IVD in focus, C7/T1 in giraffes and L4/L5 in humans—the index *i* refers to any species-specific spinal level investigated, and can here be ‘*C*7/*T*1’, ‘*L*4/*L*5’, or ‘*L*4’; for regarding the latter, see discussion, we eventually divide the respective compressive joint force *F*_*i*,||_ (Eqn 5) by the values (Table 1) of the area *A*_*i*_ of a respective adjacent vertebra endplate, as extracted from literature:(6)
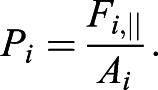


## RESULTS

**Fig. 3.**
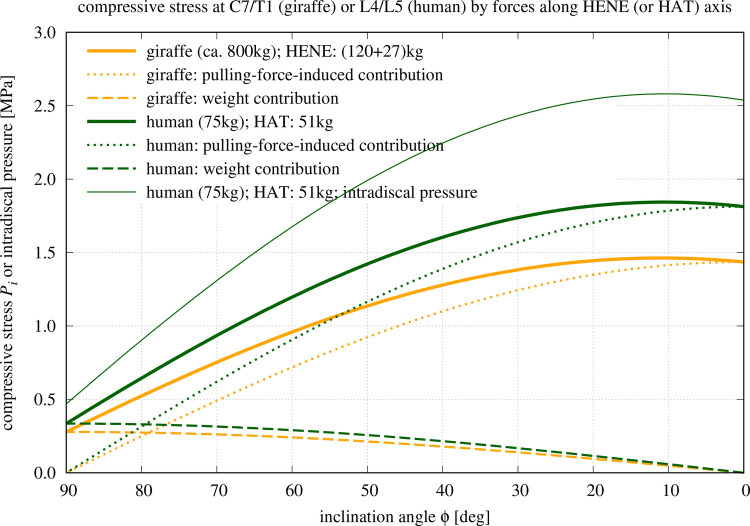
**Model-predicted compressive stress *P*_*i*_ on the IVD at the base of the giraffe's HENE (*i*=*C*7/*T*1) or human HAT (*i*=*L*4/*L*5) segments, respectively, versus forward bending (flexion) angle *φ* of the respective spinal segment, with the centre of rotation being located within the base IVD, and *φ*=90° representing ‘fully erect’.** The calculations are completely analogous to Helmuth (1985), with the only difference that we have plotted here the compressive stress (external pressure) on the segments’ base IVDs in a plane perpendicular to the longitudinal axis of the inclined spine segment (HENE or HAT). In this, we have assumed that the normal vectors of the (mean) endplates of the respective vertebrae adjacent to the base IVDs, C7/T1 in giraffes or L4/L5 in humans, respectively, point approximately along the longitudinal axis of the spine (segment). Moreover, an estimate of the correspondingly predicted human intradiscal pressure is plotted (compressive stress *P*_*L*4/*L*5_ times 1.4, Nachemson, 1960; Brinckmann and Grootenboer, 1991).

As can be seen in Fig. 3, the compressive stress values predicted by our model for the human L4/L5 IVD ranges from 0.34 MPa in fully erect posture (*φ*=90°) to a maximum of 1.85 MPa at about *φ*=10°, i.e., forward bending (flexion) of the HAT by 80°  from upright posture. For making these data immediately comparable with measured *in vivo* intradiscal pressure values (Nachemson, 1966; Sato et al., 1999; Wilke et al., 1999; Takahashi et al., 2006), we multiplied our predicted compressive stress (external pressure) values by a conservatively estimated factor of 1.4, which has been determined in *in vitro* IVD preparations (Nachemson, 1960; Brinckmann and Grootenboer, 1991). With this, we predict an intradiscal pressure of 0.47 MPa for a fully erect posture, which perfectly corresponds to three sources (Nachemson, 1966; Sato et al., 1999; Wilke et al., 1999) from which a fourth source (Takahashi et al., 2006) deviates significantly but not dramatically (0.35 MPa). At the point of maximum compressive stress, *φ*=10°, we would predict an intradiscal pressure of 2.58 MPa.

For standing with the HAT bent forward by 30° (*φ*=60°), we predict a compressive stress of about 1.2 MPa (an increase from *φ*=90° by a factor of 3.5; intradiscal pressure about 1.7 MPa). In one of the more recent studies, Wilke et al., 1999, an intradiscal pressure value of 1.1 MPa (i.e. a factor of 2.2) was directly measured for some similar forward bending (see their Table 1 and Figure 11), however, unfortunately, they did not give quantitative data of the bending magnitude. In a later paper, Wilke et al. (2001) related their 1.1 MPa value to 36°  bending, however, in terms of a local lumbar deflexion measure with poorly reported marker position referencing and angle calculation. Again differently quantifying the degree of bending, namely, by the local angular deflexion of L4 with respect to L5, Sato et al. (1999) found an increase of intradiscal pressure during some forward bending (their Table 2: 5° local L4/L5 deflexion) from 0.54 MPa to 1.32 MPa, i.e. a factor of 2.5. For forward bending of a little less than 30° from fully erect, Takahashi et al. (2006) found that their directly measured intradiscal pressure values increased by a factor of 3.7 (from 0.35 MPa to 1.6 MPa, see their Fig. 4). Finally, for sitting either relaxed upright or actively straightened, 0.46 MPa or 0.55 MPa, respectively, have been measured by Wilke et al. (1999), consistent to their data of fully erect standing. We can establish that the intradiscal pressure values predicted by our biomechanical model for quasi-static postural conditions in human L4/L5 IVDs well match the respective data directly measured *in vivo*. Our most reduced mechanical model, each just one degree of freedom and one torque-compensating, pulling-force structure, is ‘co-contraction-free’ and, hence, provides a minimum-IVD-compression estimate. Therefore, the good match with the currently available measured intradiscal pressure data proved above allows a first inference from our results: active muscular stiffness modulation that might arise from stability requirements does not seem determinative for the magnitude of compressive loads occurring in the human lumbar spine. In other words: co-contraction of muscles has minimal impact on everyday lumbar compressive load scenarios.

Both in walking (Basu et al., 2019b) and galloping (Basu et al., 2019a), giraffes hold their necks at about *φ*=35°. Values of the compressive stress on the giraffe's neck base IVD in their everyday standing postures (Fig. 3: from 0.95 MPa at *φ*=60° to 1.4 MPa at *φ*=30°) are comparable to those on the L4/L5 IVD of a human who moderately deflects its spine from its characteristic, fully erect posture (*φ*=90°), that is, within a range covering a human's daily activities. To let its compressive L4/L5 stress increase up to a giraffe's everyday maximum (1.4 MPa at *φ*=30°), the human must flex its spine forward by 39° (i.e. to *φ*=51°), with a corresponding intradiscal pressure of 1.95 MPa. The other way round, humans who carry on their everyday activities like standing, sitting, or walking in a relaxed way and close to fully erect posture, are exposed to about a third of the compressive stress values on their lumbar discs as compared with giraffes in their everyday roaming (assumed to be performed at *φ*=60°, the neutral posture; Stevens and Parrish, 2005, Fig. 6.2). The slope of the pulling-force-induced contribution to the compressive stress *P*_*i*_ treated as a function of the inclination angle *φ* (reduction means forward bending, see again Fig. 3) is proportional to the species-specific multiplier (see Eqn 5 together with Eqn 6)(7)
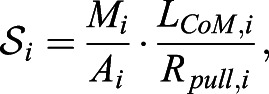
with *i* indicating the species-specific spinal level analysed, *M*_*i*_ the mass above the level, *A*_*i*_ the level's IVD area, and *R*_*i*_ the compensating structure's lever arm. This multiplier *S*_*i*_ is higher, thus, the slope steeper, in humans than in giraffes *S*_*L*4/*L*5_>*S*_*C*7/*T*1_.

## DISCUSSION

### Erecting the spine: a biomechanical design criterion

Our study has been initiated by a comparison of mechanical loads acting on comparable anatomical structures in two species. The results of the comparisons across different degrees of spine bending, which are common to the species examined, touch general evolutionary issues across all species that developed erect body parts. Namely, the degree of erectness of a species may be indicative of at least four design criteria being balanced during evolution: (1) (neural) control effort (Haeufle et al., 2014) for posture and movement (Haeufle et al., 2020), (2) loads of force-bearing structures, (3) metabolic energy consumption, and (4) functional capabilities of the motor apparatus. Two of these are definitely costs (control effort, metabolic energy), one may be indicative in itself of a balance of costs and basic limits of physics (mechanical loads), and one is a gain (motor capabilities). Criterion 1 may even be fundamental for itself, still, 1 and 3 may be closely related in some respects (Niven et al., 2007). Likewise, considering criteria 2 and 3 combined yields an example of a design trade-off. On the one hand, there are energy costs of maintaining the body material: longer lever arms would usually imply being surrounded by increased volume of other tissue to be maintained and moved. On the other hand, for both reducing structure loads and energetic costs of near-isometric force production (Taylor et al., 1980; Taylor, 1985; Kram and Taylor, 1990) longer lever arms and increased cross-sectional areas are desirable. Concerning the erection of the human spine, a specification of these criteria of evolutionary balancing would be: (1) balancing the multiple inverted (unstable) pendulum HAT against gravity likely increases the requirement for sensor deployment and their feedback signal processing. (2) The loads on the IVDs in particular must be limited by design, given that the spine is exposed to, compared to other species, increased flexibility in multiple degrees of freedom (even at once: think of throwing a stone, or a spear later in evolution) with partly high deviations from being fully erect. (3) Metabolic energy is required for continuous balancing. Also, due to working with potentially high spine deviations, muscle deployment must be limited. Realising 2 and 3 at once may have favoured ample deployment of ligaments. (4) The gain of erecting the spine is in enabling humans to carry heavy loads, combined with freeing their arms and hands from the demand to support weight, and all the potential evolutionary consequences of freed hands.

We suspended our present story at the seemingly plausible notion that erecting a part of the spine, that is, leaving a big portion of body mass (trunk or neck) from head downwards unsupported against gravity by extremities, would pose a major mechanical challenge to body material properties. As part of this notion, the challenge would grow with the size of the animal, because the ratio of the weight of the erect spine part to its supporting area might be expected to increase along with size. Our calculations have yielded an entirely different fact: erecting the cranial spine part immediately relieves the structures at its base from constantly required mechanical loads, which can be measured particularly in terms of compressive stress to IVDs. This allows reduction of the cross-sectional areas of the torque-compensating ligaments and muscles, and maybe even their lever arms if slender spine design is a goal. Additionally, the shear loads on the compressed material at the base may even be minimised. The more erect the cranial spine part, the more the pulling ligaments and muscles all along it can align with the ever-present external load due to gravity. As a consequence, the supporting weight-compensatory structures (IVDs) can be laid out to mainly resist axial compression, even contribute themselves to low-torque compensation by inhomogeneous compression, and still enable multiple degrees of spine movement by allowing moderate shear forces instead of form fitting.

We can conclude, much in line with Helmuth (1985), that erecting the cranial spine enables an animal to carry significant body-external extra load, at least if held close to the spine axis, without having to withstand higher compressive IVD stresses than when just supporting its cranial spine in a moderately forward-bent posture. Whether in a hominin or a giraffe, the compressive stress value in the spine base at fully erect posture is only about a third of the value at 30° forward flexion. In the latter neck posture, which occurs almost permanently as a giraffe's everyday biomechanical loading condition, the compressive IVD stress is about 1 MPa. Using empirical data in humans, this value would correspond to an intradiscal pressure value of about 1.4 MPa, which can, hence, be considered a non-critical norm for repeatedly occurring everyday activities of terrestrial vertebrates. For comparison with a compressive stress value of 1 MPa, Alexander (1985) estimated dinosaurs’ spinal everyday stress values to range from 2 MPa to 3 MPa, and Morris et al. (1961) calculated that stress extremes as high as 6.2 MPa can be reached by humans in weight lifting.

In a nutshell, erecting the spine definitely opens, from a basic mechanical point of view, an enormous potential to carry extra loads. The major biomechanical design and movement challenges then are, of course, to handle such extra loads, that is, to ideally bring them into carrying positions as close to the spine axis as possible, to actively balance the trunk itself plus the extra load in unstable upright postures, and to discharge the extra load, as for these tasks the spine must be somehow deflected from the upright position, which may definitely and immediately increase body-internal loads beyond critical material limits.

### Morphological examinations: the significance of knowing anatomical and material properties well

Another main conclusion of our reductionist, biomechanical model calculations, is that the geometry, lever arms and IVD areas (see Eqn 7 and Eqn 5, with Eqn 6) of the main load-bearing body-internal structures (muscles, ligaments, IVDs, and vertebrae), strongly dominates the magnitude of the compressive stress on the IVDs in the spine, as soon as the spine is deflected from full erection. Already at 30° deflection from full erection, the pulling-force-induced contribution to compressive stress has increased to more than two times HAT or HENE weight, and to more than three times the weight contribution at this deflection, as the weight contribution progressively decreases with deflection (Fig. 3). This conclusion applies to as much the stress values occurring in the real, biological world as predictions based on a model. By all means, it seems biomechanists agree that local load distributions between various load-bearing body-internal structures somehow depend on the local structures’ anatomical and material properties. Using intradiscal pressure values as one example of how much internal loads depend on such properties, this study, in combination with two similar ones (Helmuth, 1985; Jäger and Luttmann, 1989), can be understood as highlighting the sensitivity of internal loads with respect to particularly anatomical parameters.

For example, a human's mean lever arm for a characteristic structure pulling past the L4/L5 IVD was assumed in these three studies: 0.049 m here, 0.05 m in Helmuth (1985), and 0.065 m in Jäger and Luttmann (1989). Thus, the data in the latter study deviate by about 20%, which is immediately reflected by differences between our Fig. 3 and their calculated compressive IVD stress values. Just one further model parameter included (here) or neglected (there), namely the transmission factor of about 1.4 (Nachemson, 1960; Brinckmann and Grootenboer, 1991) from (external) compressive stress to intradiscal pressure values, amplifies to well over 40% lower pressure predictions by Jäger and Luttmann (1989, Fig. 7) than by us. Using the length of the spinous process as a caliper, the length of the lever arm of this or that ligament or muscle passing the IVD can vary by at least a factor of three between maximum and minimum possible values; with this, the magnitude of the torque contribution of a respective structure may be uncertain accordingly. However, as all pulling structures have to share the limited anatomical space, the assumption of a net lever arm length with a corresponding net pulling force value will, thus, be uncertain on a similar level only at low forces. With the force level increasing with forward flexion, the uncertainty of the predicted pulling force and, thus, the compressive stress will largely diminish, as all structures are likely to contribute, which yields an averaging effect regarding lever arms.

Another example of model-based predictions of compressive stress values in the base IVD area of hominins, namely, australopiths had been provided by Helmuth (1985). At 30° forward flexion (i.e. *φ*=60°), he gave calculated values in the range from 3.8 MPa to 7.5 MPa in his paper text, which would be 3.2 to 6.3 times higher than predicted by us for a human. A little confusing, taking the morphometric data of the biggest australopith specimen considered by Helmuth (1985) (*Australopithecus boisei*: body height 1.63 m, body weight 50 kg, thus, *M*_*HAT*_=34 kg), as well as both the highest lever arm *R*_*pull*_=3.7 cm and the exactly human-like *L*_*CoM*,*HAT*_=0.265 m given there, our re-calculation yielded compressive stress values of only 2.4 to 3.3 times those in humans, i.e., 2.9 MPa to 4.0 MPa at *φ*=60°. These compressive stress ranges are mainly due to the range of endplate areas, 3.6 cm^2^ to 5.2 cm^2^, given by Helmuth (1985), which had been extracted there from even earlier australopith literature (Johansson et al., 1982). As these values are much lower than in a human (about 15 cm^2^), the compressive stress values calculated so far have been so much higher in australopiths than in humans. Using the area values above according to Helmuth (1985); Johansson et al. (1982), at fully erect posture (*φ*=90°), our re-calculated compressive stress values would range from 0.65 MPa to 0.92 MPa. Assuming the lowest lever arm value *R*_*pull*_=2.6 cm, we would even predict 0.93 MPa to 1.31 MPa, very close to giraffes’ everyday compressive stress values.

However, more data on australopiths have been collected since 1985. From the calibrated Figure 7.14 in a more recent source (Williams and Meyer, 2017), the dimensions of an *Australopithecus afarensis* L4 endplate can be read: a width of *W*_*L*4_=4.8 cm and a depth of *D*_*L*4_=2.8 cm, i.e. an estimated area of *A*_*L*4_=10.6 cm^2^. With these data, *R*_*pull*_=3.7 cm, and again the (likely adult) australopith body height and weight assumed above, we eventually predict practically human-like compressive stress values of 0.32 MPa at *φ*=90° and 1.40 MPa at *φ*=60° in adult australopiths.

Accordingly, properly determining endplate areas, just like muscle and ligament lever arms, in extant species or from fossil records are all key to well predicting compressive IVD stresses. As a last example, referring to material properties, it has recently been shown that widely scattering published data on spinal ligament properties, their stiffnesses (Mörl et al., 2020; Damm et al., 2019) and rest lengths (Mörl et al., 2020) in particular, are a source of erroneous calculations of loads on all spinal structures. Consequently, our second major conclusion is that the mechanical function(s) of an anatomical structure, as well as functional interrelations between structures, should always be kept in mind when experimentally examining and determining anatomical and material properties.

Our simple model for calculating compressive stress does only factor in a lumped anatomy of all force generators passing the IVD and the IVD's endplate area, but no further geometrical, physical, or physiological knowledge of the load-bearing structures surrounding the IVD. Nevertheless, it can reliably predict the compressive stress on an IVD, because, among other things, this is the only joint structure that is compressed in flexion, and the compressive stress acting on it, just like its intradiscal pressure, intrinsically represents an accumulative load quantity. However, to eventually understand, for example, damage to the load-bearing structures occurring during movement, it seems almost trivial to punctuate that structure-resolved modelling is absolutely essential. Even more, and in the spine in particular, the structures’ non-linear interactions determine the load distribution among them (Mörl et al., 2020). Thus, to resolve cause–effect chains, that is, to causally understand natural processes, structure-resolved, mechanistic models are indispensable, beyond a simple reductionist approach like the present.

### Distribution among passive and active pulling forces in the (human) spine

Accordingly, let us have a closer look at our present calculation results (for humans) with the help of a complex model of the human lumbar spine that has incorporated all of the main spinal load-bearing structures (Mörl et al., 2020). We verify the consistency of the load predictions by both models, and deploy the complex model as a hand lens for decomposing the net structural pulling force in our present, simple model into force contributions by the main load-bearing structures. States of spine flexion in this complex, structure-resolved model can be immediately compared with corresponding states of the present reductionist model, because model values of body height and weight are similar. Whereas the spine flexion is gravity-induced in the simple model here, a torque generated by a simulated machine motor flexed the complex model of a human subject lying on its side, so that weight did not add a compressive force to the spine.

**Fig. 4.**
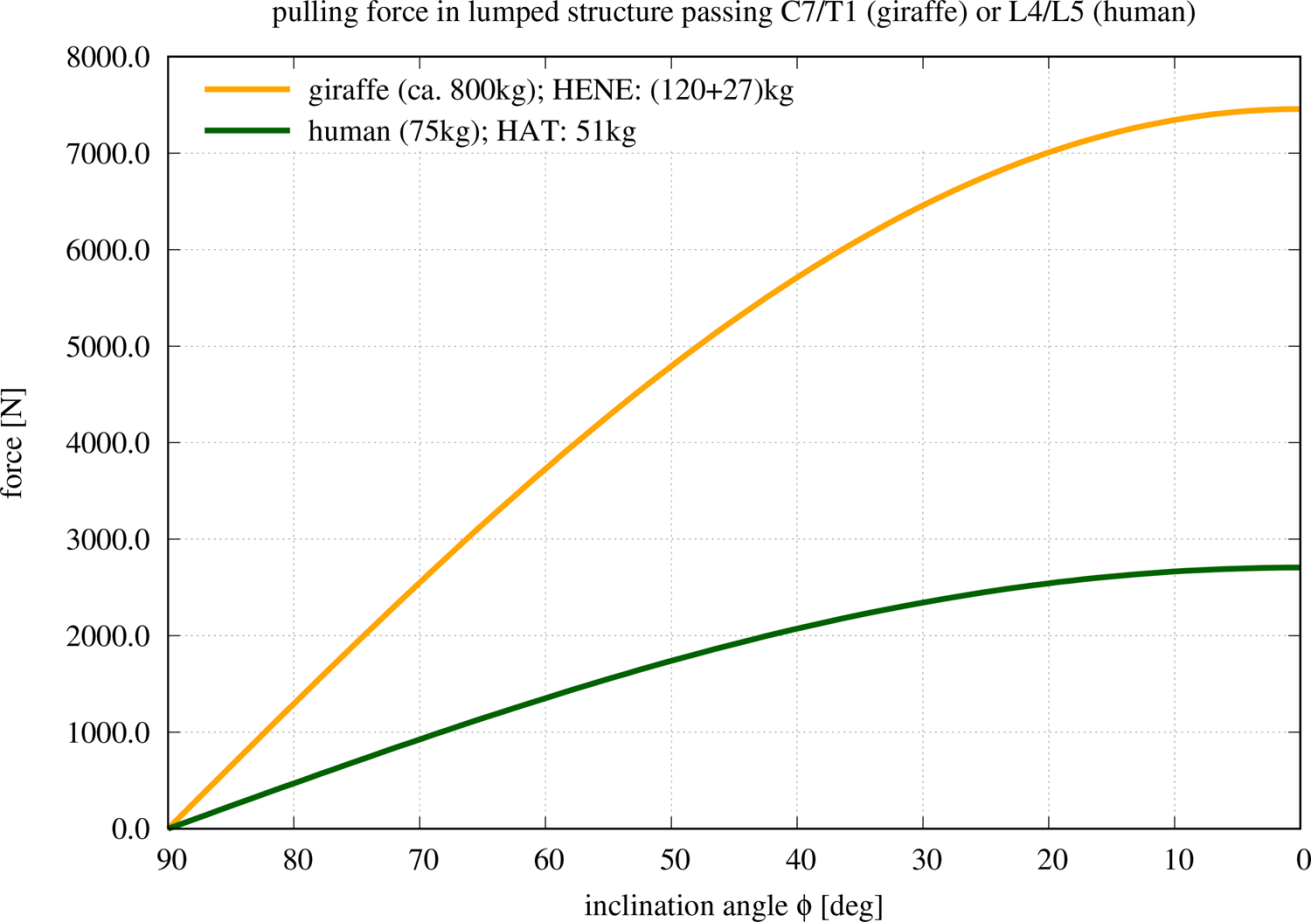
**Model-predicted force in the lumped torque-generating structure pulling past the IVD at the base of the giraffe's HENE or human HAT segments, respectively, versus forward bending (flexion) angle** *φ* **of the respective spinal segment, with the centre of rotation being located within the base IVD, and *φ*=90° representing ‘fully erect’.** The lengths of the respective lever arms are compiled at the bottom of Table 1, and multiplying the respective lever arm value with the force in this plot immediately yields the corresponding joint torque value.

The point of comparison shall be the state of maximum spine flexion in the complex model, namely, its (steady) flexion response at about 30 N m IVD joint torque. This torque value occurs in our present model when the spine is bent forward by 13° from upright posture (*φ*=77° with 
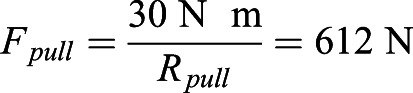
 from Fig. 4 and *R*_*pull*_=0.049 m from Table 1). At this 30 N m point, a compressive stress of 0.73 MPa (Fig. 3) is predicted by the present model, while the directly weight-induced contribution is 0.33 MPa. At about the same 30 N m IVD torque in the complex model (Mörl et al., 2020, Fig. 5), a compressive IVD force of almost exactly 500 N has been predicted (Mörl et al., 2020, Fig. 6), which corresponds to 0.33 MPa if a mean endplate area of 15 cm^2^ (Table 1) at L4/L5 level is assumed. Adding 0.33 MPa due to the weight of our HAT mass (51 kg) for comparison with our present model prediction, 0.66 MPa would be expected. The difference between 0.66 MPa and 0.73 MPa can be largely explained. As predicted by the complex model, 75% of the IVD joint torque is caused by forces due to structures (passive muscles and ligaments) pulling past the IVD (Mörl et al., 2020, Fig. 5), the remaining 25% are generated by the squeezed IVD itself. If, accordingly, this 25% torque contribution by the IVD itself were non-existent and had instead to be additionally generated by structures pulling past the IVD, the compressive force would be roughly a third higher than 500 N, therefore, the corresponding compressive stress induced by these forces would be about 0.44 MPa, and adding 0.33 MPa due to bearing HAT weight would yield 0.77 MPa, which is very close to 0.73 MPa predicted by the present, simple model. It has also been found by Mörl et al. (2020) that ligament forces in particular are certainly still moderately over-estimated by the complex model. Thus, the 500 N compressive force predicted by the complex model, and 0.77 MPa compressive stress with it, still have to be taken with some care. In any case, the consistency of load predictions in the human lumbar spine by our present, simple model and a much more complex one is high. Any co-contraction of pulling structures will, of course, increase the compressive stress on the IVD. Therefore, the compressive stress values calculated by our simple model approximate the minimum to be expected from basic mechanics. If the values found in nature are indeed measured to be close to this minimum, this may be an indication that co-contraction in the human spine has been avoided as far as possible during its evolutionary design, with the minimisation of co-contraction entailing minimised metabolic energy consumption.

By means of our present, simple model, we predict the maximum compressive stress at the base of HAT or HENE, respectively, to occur if they are bent forward by 80° from upright posture (*φ*=10°; Fig. 3), and the maximum torque-compensating pulling force is always required when being bent fully forward (*φ*=0°; Fig. 4). In humans, for example, the corresponding compressive stress, IVD joint torque, and pulling force values are predicted as 1.81 MPa, 132.6 N m, and 2706 N (Fig. 4), respectively, at *φ*=0°. Gracovetsky (1986) estimated the maximum isometric force of all human lumbar back muscles to be about 2500 N. A more recent study documented a mean cross-sectional area of 87.4 cm^2^ of all lumbar muscles in men (Chang et al., 2016). The mean maximum isometric stress of skeletal muscle at 37°C is about 25 N cm^−2^ (e.g. Mörl et al., 2020, section 2.5.7), which would result in an even lower maximum isometric force of 2185 N. There are, thus, strong indications that the lumbar muscles alone would not even be sufficient for a human to lift its own upper body masses, and certainly not external loads, at least if markedly bent forward but still straightened. Muscles not located in the lumbar region, but reaching into it via an aponeurosis (e.g. actuated by m. latissimus), may aid in load lifting.

In any case, other passive pulling force generating structures like ligaments or the torque-generating annulus fibrosus are essential for spinal functioning. In giraffes, there is, likewise, a strong passive force involvement, and probably even stronger than in humans, as can be inferred from giraffes’ almost non-curved neck at a neutral posture (Christian and Dzemski, 2007; Stevens and Parrish, 2005) of about *φ*=60° (Stevens and Parrish, 2005, Fig. 6.2), and a marked nuchal ligament (Jouffroy, 1992; Taylor and Wedel, 2013), which together make it very likely that muscle activity is required to lower the head down to toe level for drinking water (referred to as ‘browse by ventriflexion’ in Stevens and Parrish, 2005). In humans, passive structures resist already moderate flexion of the lumbar spine (Mörl et al., 2020). Holding significant parts of body mass in positions that are not fully erect, like in everyday postures of giraffes, permanently requires static compensating pulling forces. Perfectly upright postures, like in hominins, seem to be clearly less or non-demanding. However, balancing these masses around full erection comes with the demand of struggling with the inherent instability by constantly and dynamically loading and unloading the compensating structures during all phases of everyday activity. Either way, fully erect or not, using active muscles for generating compensatory forces is metabolically demanding. Therefore, relying strongly on passive compensatory contributions seems to be an appropriate design by nature for balancing erect body portions.
